# Reproductive function and behaviors: an update on the role of neural estrogen receptors alpha and beta

**DOI:** 10.3389/fendo.2024.1408677

**Published:** 2024-06-24

**Authors:** Thomas Torres, Nolwenn Adam, Sakina Mhaouty-Kodja, Lydie Naulé

**Affiliations:** Sorbonne Université, CNRS UMR8246, INSERM U1130, Neuroscience Paris Seine – Institut de Biologie Paris Seine, Paris, France

**Keywords:** estrogen receptors, hypothalamic-pituitary-gonadal axis, reproductive behaviors, sex steroids, neuroendocrinology

## Abstract

Infertility is becoming a major public health problem, with increasing frequency due to medical, environmental and societal causes. The increasingly late age of childbearing, growing exposure to endocrine disruptors and other reprotoxic products, and increasing number of medical reproductive dysfunctions (endometriosis, polycystic ovary syndrome, etc.) are among the most common causes. Fertility relies on fine-tuned control of both neuroendocrine function and reproductive behaviors, those are critically regulated by sex steroid hormones. Testosterone and estradiol exert organizational and activational effects throughout life to establish and activate the neural circuits underlying reproductive function. This regulation is mediated through estrogen receptors (ERs) and androgen receptor (AR). Estradiol acts mainly via nuclear estrogen receptors ERα and ERβ. The aim of this review is to summarize the genetic studies that have been undertaken to comprehend the specific contribution of ERα and ERβ in the neural circuits underlying the regulation of the hypothalamic-pituitary-gonadal axis and the expression of reproductive behaviors, including sexual and parental behavior. Particular emphasis will be placed on the neural role of these receptors and the underlying sex differences.

## Graphical Abstract

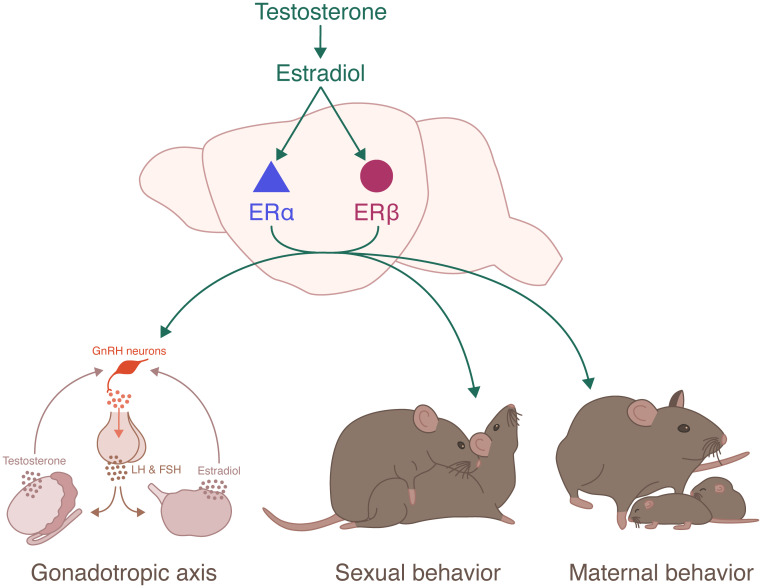

## 1 Introduction

In mammals, fertility allows the perpetuation of the species. Fertility relies on a fine regulation of reproductive function involving both adequate neuroendocrine regulation of the hypothalamic-pituitary-gonadal (HPG) axis, and synchronized expression of male and female sexually dimorphic reproductive behaviors.

**Figure 1:**
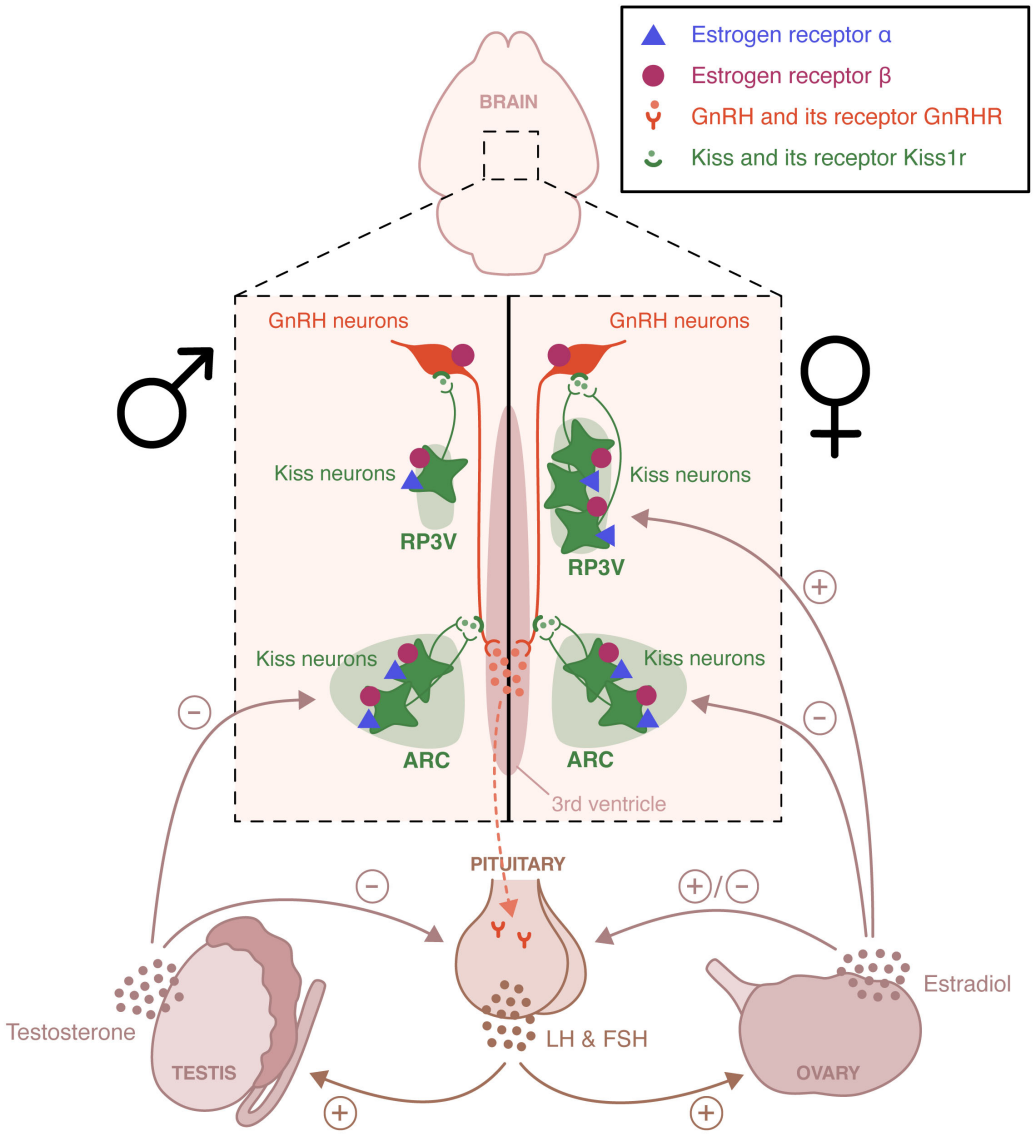
Schematic representation of the neural circuits involved in the estradiol regulation of the hypothalamic-pituitary-gonadal axis. Estrogen receptor (ER) α and ERβ are expressed in the rostral periventricular area of the third ventricle (RP3V) and the arcuate nucleus (ARC) of both males and females. These two hypothalamic nuclei are essential to the control of estradiol positive and negative feedback, respectively. The loss of the positive feedback in males is an example of sexual dimorphism. ERβ, unlike ERα is also expressed in gonadotropin-releasing hormone (GnRH) neurons and appears to participate to the pubertal activation of the hypothalamic-pituitary-axis. LH, Luteinizing hormone; FSH, follicle-stimulating hormone.

Within the HPG axis, the pulsatile release of gonadotropin releasing hormone (GnRH) from hypothalamic GnRH neurons in the hypothalamic-pituitary portal system activates the neuroendocrine secretion of the gonadotropins luteinizing hormone (LH) and follicle-stimulating hormone (FSH) (**Figure 1**). LH and FSH stimulate the gonads and trigger gametogenesis and secretion of gonadal steroid hormones. Sex steroid hormones in turn exert a feedback control on the HPG axis (1). In males, testicular testosterone exerts a negative feedback control on hypothalamic GnRH and pituitary LH release (2). In females, estradiol negatively regulates hypothalamic GnRH secretion for most of the estrous cycle, except during the proestrus phase. During this phase, the growth of ovarian follicles is associated with a rise in circulating estradiol concentration that exerts hypothalamic and pituitary positive feedbacks, leading to the LH discharge necessary for ovulation and induction of female receptivity (3, 4). GnRH neurons activity is finely regulated by a complex neural network including kisspeptin-, neurokinin B-, glutamate- and GABA-expressing neurons. Glial cells, especially astrocytes and tanycytes, also participate in this regulation (5). Kisspeptin neurons are located in two distinct hypothalamic regions, the arcuate nucleus (ARC) and the rostral periventricular area of the third ventricle (RP3V). ARC kisspeptin neurons, referred to as KNDy neurons because of their coexpression of kisspeptin, neurokinin B and dynorphin, participate in estradiol negative feedback and coordination of GnRH pulses (6). RP3V kisspeptin neurons are essential to the regulation of estradiol positive feedback driving the generation of the female LH surge (7). This positive feedback is lost in males. It is a primary example of sexual dimorphism.

**Figure 2:**
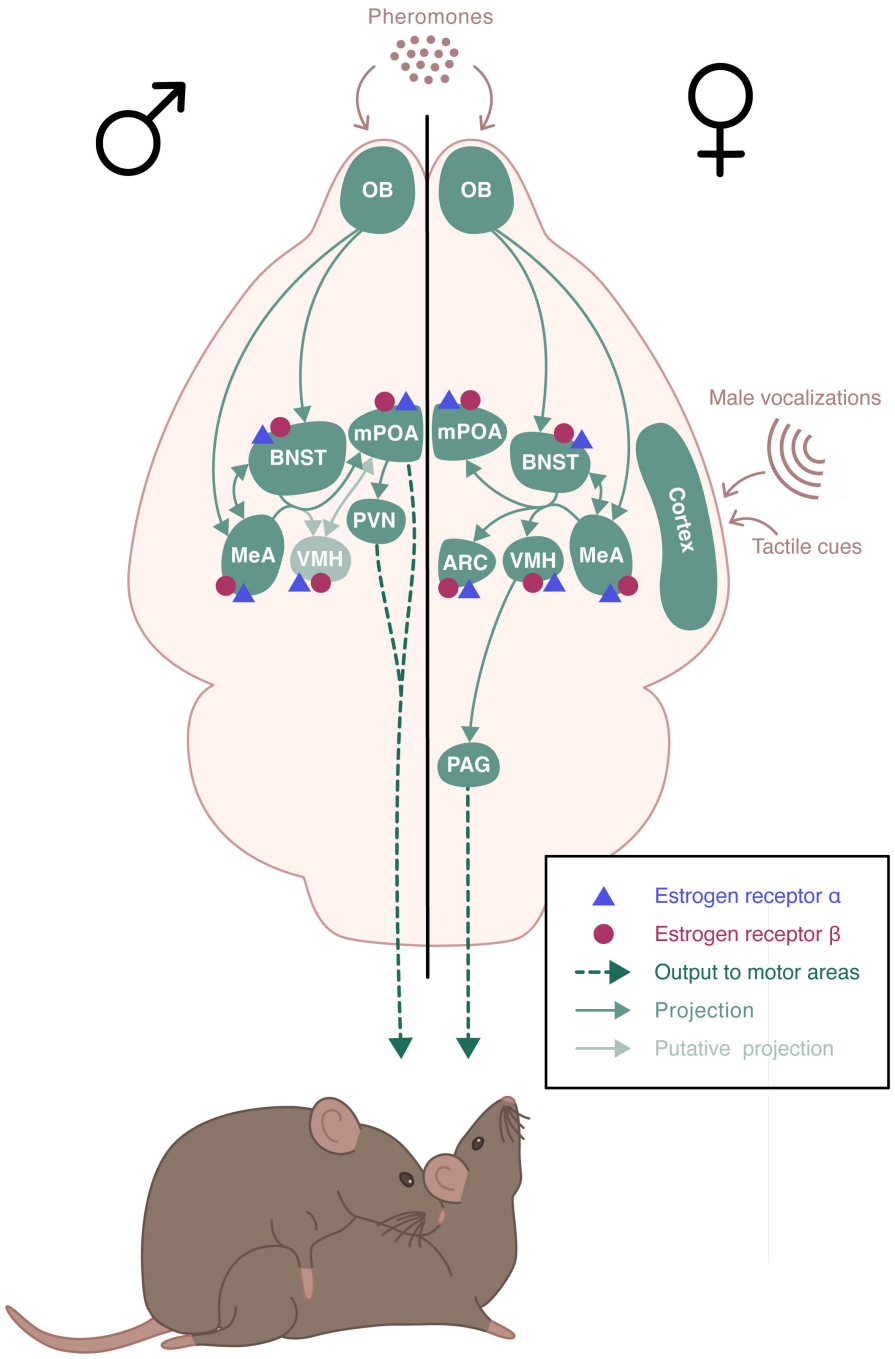
Schematic representation of the neural circuits involved in the estradiol regulation of sexual behaviors. Estrogen receptor (ER) α and ERβ receptors are expressed throughout the neural circuit involved in the expression of sexual behaviors in a sexually dimorphic manner. Sexual behavior is triggered by pheromones that are detected by chemosensory neurons. These neurons project to the olfactory bulb (OB) which innervates the medial amygdala (MeA) and the bed nucleus of the stria terminalis (BNST), which send projections to the medial preoptic area (mPOA) and the ventromedial nucleus of the hypothalamus (VMH). In males, mPOA plays a critical role in the expression of sexual behavior. Projections from the hypothalamic paraventricular nucleus (PVN) are sent to the spinal centers that promote erection and ejaculation. In females, VMH is essential for the activation of sexual behavior. It sends projections to the periaqueductal gray matter (PAG) that project to the brainstem and spinal cord, which innervate the axial muscles involved in the lordosis posture. There is also an inhibitory circuit for lordosis behavior involving the ARC and the POA.

In addition to a functional HPG axis, fertility requires appropriate and optimal display of reproductive behaviors, including sexual and parental behavior. Sexual behavior is sexually dimorphic. In rodents, it includes an appetitive phase, during which both sexes actively stimulate their partners by releasing pheromones and displaying appetitive behaviors. These behaviors include ano-genital investigations, series of approaches and solicitations, as well as male emission of ultrasonic vocalizations. These are followed by a consummatory phase, where male mounts are associated with female display of the lordosis posture, favoring intromission (8). Males express a continuous sexual activity, while females are only receptive during the proestrus phase of the estrous cycle, after the sequential rise in estrogen and progesterone (9). Sexual behavior relies on the activation of complex, sexually dimorphic neural circuits (10)(**Figure 2**). It is triggered by the detection of sensory stimuli, especially pheromones, that are detected by chemosensory neurons located in the vomeronasal organ and the main olfactory epithelium (11, 12). These neurons project to the main olfactory bulb (MOB) and the accessory olfactory bulb (AOB), which innervates among other structures, the medial amygdala (MeA) (13, 14). Neurons from the MeA then project to the bed nucleus of the stria terminalis (BNST), the medial preoptic area (mPOA), and the ventromedial nucleus of the hypothalamus (VMH). In males, the mPOA plays a critical role in the expression of sexual behavior. Projections from the hypothalamic paraventricular nucleus (PVN) are sent to the spinal centers that promote erection and ejaculation (15, 16). In females, the VMH is essential for the activation of sexual behavior. It sends projections to the periaqueductal gray matter (PAG) that project to the brainstem and spinal cord, which innervate the axial muscles involved in the lordosis posture (17). There is also an inhibitory circuit for lordosis behavior involving the ARC and the POA (18).

**Figure 3:**
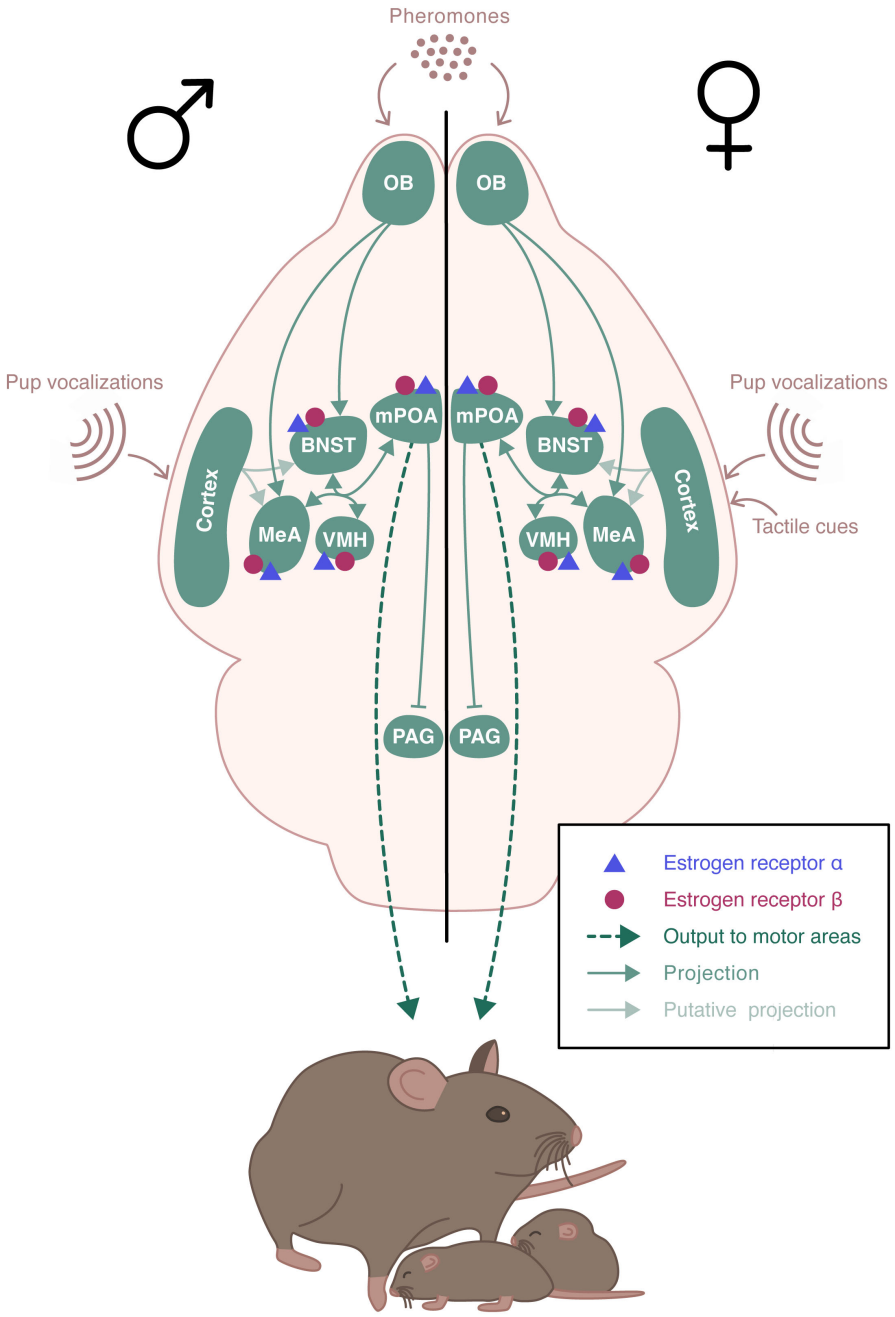
Schematic representation of the neural circuits involved in the estradiol regulation of parental behavior. ERα and ERβ receptors are expressed in the brain regions involved in the expression of parental behavior. Pup’s sensory information is integrated into a neural circuitry that includes the medial amygdala (MeA) and the bed nucleus of the stria terminalis (BNST). These regions project to the medial preoptic area (mPOA) which send projections to various downstream regions including the hypothalamic paraventricular nucleus (PVN) and regions of the dopaminergic system. The neural pathway underlying paternal behavior remains unclear, but it seems to be similar to the one for maternal behavior, with mPOA as a key region.

Parental behavior occurs primarily in females and is minimal or absent in males of many mammalian species. In rodents, parental behavior is exhibited by the mother, with the exception of few species that display biparental care, such as the prairie vole or the California mouse (19). Parental behavior defines all behaviors exhibited to increase pups’ chances of survival and development (20). Typically, rodents tend to avoid pups, or even exhibit aggressive behavior and infanticide towards them. A behavioral “switch” occurs in the early days of gestation, with females displaying an intensive nest building and increased aggressivity towards intruders. At birth, dams show a strong interest in newborns and become highly responsive to gustatory, olfactory, and tactile cues. Dams also rapidly respond to ultrasonic vocalizations emitted by pups, by retrieving them safely to the nest (21, 22). Pup’s sensory information is integrated into a neural circuitry that includes the MeA and the BNST (**Figure 3**). These regions project to the mPOA that promotes pup attractivity. In turn, the mPOA projects to various downstream regions that facilitate the establishment and maintenance of maternal behavior, including the PVN rich in oxytocinergic neurons, and the ventral tegmental area (VTA) of the dopaminergic system (23). Males and virgin or non-lactating females of uniparental species can also exhibit parental behavior. This occurs spontaneously in most strains of laboratory mice, although the level of pup care is much lower than that given by dams (24). The neural pathway underlying paternal behavior seems to be similar to the one for maternal behavior, with mPOA as a key region (19).

The reproductive function and behavior are tightly regulated by sex steroid hormones. The sexually dimorphic hormonal control of mating was first demonstrated by the pioneer work of Phoenix and collaborators in 1959 (25). This study, and those that followed, demonstrated the existence of both organizational and activational effects of sex steroids (26). Androgens and estrogens act at specific periods of development to organize, in a sex-dependent manner, the neural circuits controlling reproductive function and behavior, which are then activated by sex steroids in adulthood.

Organizational changes occur during sensitive periods of development. Although initially assumed to affect only males during perinatal life, increasing evidence supports that organization takes place in both sexes at multiple developmental windows, including the perinatal, postnatal, and pubertal periods. It is well known that perinatal masculinization of the male brain results from two testosterone bursts occurring before and after birth. In addition, brain feminization in females appears to occur later as circulating estradiol increases during postnatal and prepubertal development (27–31). These organizational and activational effects of testosterone and estradiol are mediated through their receptors: estrogen receptors (ERs) and androgen receptor (AR). In males, testosterone can also be aromatized due to neural expression of cytochrome p450 aromatase into neural 17β-estradiol, which then acts on ERs (32, 33).

The present review focuses on the neural role of the ERs in male and female reproduction (for a review of the role of AR in the central nervous system see (34)). In particular, we will review genetic evidence using transgenic mouse models and recent technological tools that have provided a better understanding of the role of ERs in estradiol-induced regulation of the HPG axis and reproductive behaviors.

## 2 Estrogen receptors: types and neural expression

In the mouse, ERα (66 kDa) and ERβ (54 kDa) are encoded by estrogen receptor 1 (*Esr1*) and estrogen receptor 2 (*Esr2*) genes located on chromosomes 10 and 12, respectively (35, 36). These receptors belong to the nuclear receptor superfamily and act as transcription factors. The primary regulation of gene expression involves direct binding of the receptor to EREs sequences as it is the case for example for progesterone receptor (*Pgr*) (37), Kiss-1 metastasis suppressor (*Kiss1*) (38), dynorphin (*Dyn*) (39) and brain derived neurotrophic factor (*Bdnf*) (40). In addition to its slow genomic action, rapid effects of estradiol have been observed. These nongenomic effects are generally initiated by the binding of estradiol to membrane receptors including the classical ERs and other receptors such as estrogen receptor alpha delta 4 (ERαΔ4), membrane estrogen receptor G alpha q (mER-Gαq), G protein-coupled receptor 30 (GPR30), estrogen receptor X (ER-X) and saxitoxin binding protein (STXBP) (41). Binding of estradiol to these receptors activates intracellular signaling pathways such as the mitogen-activated-protein kinase (MAPK), protein kinase C (PKC), protein kinase A (PKA), or phosphotidyl inositol 3 kinase (PI-3K) pathways (42). Mechanisms by which these membrane ERs (mERs) activate these pathways remain unclear. For example, in female rats, mER interaction with membrane metabotropic glutamate receptors (mGluR) was shown to participate in lordosis behavior, through μ-opioid receptor internalization and activation of the fast calcium response, and could also affect long term genetic expression via CREB phosphorylation (43–46). The present review focuses on nuclear ERα and ERβ given their critical and well-identified roles in male and female reproduction.

ERα and ERβ are expressed throughout the neural circuits that control reproductive function and behavior. In adult mice, *Esr1* mRNA and protein have been detected in the BNST, VMH, mPOA and ARC (47–49). *Esr2* mRNA is also present, less abundantly than *Esr1*, in the VMH, mPOA and ARC (50–52). Given the lack of selectivity of anti-ERβ antibodies (49, 53), several transgenic ERβ-enhanced green fluorescent protein (EGFP)/-red fluorescent protein (RFP) mouse lines were used and confirmed that the localization of the ERβ protein was similar to that of the transcript (48, 54, 55). During the development, ERα and ERβ are present in the mouse nervous system as early as embryonic day (E) 13 (56). ERα and ERβ mRNA and protein levels change considerably during development in a regional and sex-specific manner. For example, in the anteroventral periventricular nucleus (AVPV), the number of ERα-expressing cells decreases from postnatal day (PND) 0 to PND14 in females, then increases with age, in contrast to males, which show no significant variations over time. In this region, the number of ERβ-expressing cells is highest at PND0 in both sexes and decreases with age in males but not in females (54, 55). In the VMH, ERα-expressing cells are present in both sexes with maximal expression at PND0, followed by a reduction from PND0 to PND14. Then, females show an increase in expression at the end of the pubertal period (PND42-PND56). In males, ERα-expressing cells remain low, and are therefore lower than in females. The number of ERβ-expressing cells in the VMH is also highest at PND0 for both sexes, but more important in females than in males at PND0 and PND7. A sharp decrease is then observed in both sexes that abolishes sex differences after PND7 (54, 55). In the BNST, a greater number of ERα-expressing cells has been described in adult female compared to male mice (57). Indeed, ERα-expression increases in female during development, while remaining constant in males (except for a transient increase at PND28). ERβ-expressing cells remains constant in females, whereas it gradually increases in males to reach its highest level at PND42 and PND56 (55).

## 3 ERα versus ERβ in estradiol-induced regulation of the hypothalamic-pituitary-gonadal axis

Many studies have focused on the sites of action of estradiol in the regulation of the HPG axis. Does it act directly on GnRH-expressing neurons or indirectly via other cells within the GnRH network? This question was raised by data showing that GnRH neurons do not express *Esr1*. Of note, they also do not express *Ar*. Nevertheless, these neurons do express *Esr2* and present detectable levels of ERβ protein in mice, rats, and humans (58–62) (**Figure 1**).

### 3.1 Role of ERα in the regulation of the HPG axis

#### 3.1.1 Models of ubiquitous *Esr1* deletion

Mouse models with ubiquitous deletion for *Esr1* (ERαKO) were obtained by homologous recombination targeting exon 2 (63) or exon 3 (64–69). ERαKO males showed a structurally normal urogenital tract, but with a strong decrease of testis weight and sperm count (63, 70). Females ERαKO uterus and vagina were hypoplastic with no cyclic morphological changes. They exhibited a polycystic ovary phenotype with an absence of corpora lutea, and anovulation (66). Both ERαKO males and females were infertile (63–66, 70). Circulating levels of testosterone were slightly increased in ERαKO males but circulating LH levels were unchanged showing no alteration of the negative feedback (70–72). In contrary, ERαKO females showed no negative or positive feedback of estradiol. Indeed, basal LH level was greatly increased in intact ERαKO female, and estradiol treatment in ovariectomized ERαKO females did not trigger the LH surge, unlike wildtypes (68, 69, 72–75).

The role of non-classical ERα signaling pathways was also investigated using a mouse model carrying a mutation in the ERα DNA recognition sequence that abolishes ERα signaling through ERE binding mechanisms (ERα^+/AA^ animals (76);). ERα^+/AA^ males were fertile and showed normal testosterone levels suggesting that ERE-independent ERα signaling is sufficient for some male reproductive function (77). Mutant females, however, were sterile due to uterine defects and anovulation (76). A knocking mutant allele model that selectively restores ERE-independent signaling in ERαKO mice (ERα^-/AA^ animals) revealed that these signaling were able to partially restore estrogen negative feedback on LH secretion, but were not sufficient to mediate estrogen positive feedback, increase GnRH neuron firing to generate the LH surge or mediate spontaneous ovulation (75, 78).

#### 3.1.2 Models of brain specific *Esr1* deletion

ERα is thus essential for fertility in both sexes and required for both estradiol positive and negative feedback in females. Nevertheless, ERα is present in many peripheral tissues including male and female reproductive tracts, thus the effects described in ERαKO animals, especially the infertility, do not allow to distinguish between neural and peripheral effects. Studies using more restricted deletion models are reported below.

A mouse line selectively deleted for *Esr1* in forebrain neurons (including in the cortex, hippocampus, BNST, amygdala, olfactory bulb, striatum, thalamus and hypothalamus) and pituitary was generated by crossing mice carrying loxP sites on either side of exon 3 of *Esr1* with mice expressing Cre recombinase under the control of the calcium/calmodulin-dependent protein kinase IIα promoter (ERα^fl/fl^; CamKIIα-Cre) (69). Both male and female mutants were infertile. ERα^fl/fl^; CamKIIα-Cre females showed strong abnormalities of their reproductive organs, an absence of estrous cycle and defect in ovulation (69, 79). Similar to ubiquitous ERαKO mice, estradiol injection in mutant females did not trigger the LH surge (69). Basal LH level of ERα^fl/fl^; CamKIIα-Cre female was no different from that of control females, but mutant mice did not show an increase in circulating LH levels after ovariectomy (79). The importance of ERα in adulthood in this negative feedback was revealed using an inducible tamoxifen-based Cre-LoxP model, which triggered more than 50% reduction in the number of ERα-expressing cells (79). This treatment resulted in lack of estrous cyclicity and disruption of negative feedback. Indeed, the increase in LH observed after ovariectomy was decreased in mutant mice, and estradiol treatment failed to restore baseline LH levels. These results revealed the importance of ERα in forebrain/pituitary in regulating both positive and acute negative feedback of estradiol.

Deletion of *Esr1* selectively in neural cells was generated by crossing ERα^fl/fl^ females with males expressing the Cre recombinase under the control of the promoter and nervous system-specific enhancer of nestin (ERα^Nescre^ (80)). This model allowed the deletion of *Esr1* in both neuronal and glial cells but not in the pituitary. Females ERα^Nescre^ presented early puberty initiation with advanced vaginal opening and first estrus. In adulthood, mutant females showed a diminution in ovary weight with absence of corpora lutea and estrous cycle arrest. This was accompanied with a decrease in the number of kisspeptin cells in the RP3V in females. An increase in uterine weight was also reported, related to elevated levels of circulating estradiol in mutant females, similar to what has been observed in other ERαKO mouse model. In adult males, seminal vesicles weight and testosterone level were increased in mutant mice compared to controls, but no modification in testis weight was observed. In contrary to females, mutant males were fertile. Thus, while neural ERα is critical for normal cyclicity, fertility and regulation of both estradiol positive and negative feedback on LH secretion, sex differences are observed, with a more critical role in females than in males. Several studies, described below, aimed to decipher the specific cell population and neural circuits involved in ERα-mediated regulation of estradiol. Because ERα is not present in GnRH neurons, estradiol appears to act indirectly via afferent circuits.

Kisspeptin neurons have been widely studied for their potent activation of GnRH neurons and are known to be regulated by estradiol through ERα. ERα is expressed in around two-third of RP3V kisspeptin-expressing cells and nearly all ARC kisspeptin cells (81). Estradiol inhibits kisspeptin expression in the ARC while activating its expression in the RP3V (38, 82). This opposing regulation of estradiol on kisspeptin expression was abolished in ERαKO mice (38). To investigate further, a mouse model to delete *Esr1* specifically in kisspeptin expressing cells (KERKO) was generated by crossing mice floxed for exon 3 of *Esr1* with mice expressing Cre recombinase under the control of kisspeptin promoter (81, 83, 84). Mice carrying this mutation showed advanced vaginal opening, disruption of ovarian cyclicity, complete absence of corporal lutea leading to infertility. Adult females KERKO exhibited a reduced LH secretory response to ovariectomy compared to control suggesting an alteration of the estradiol negative feedback. Nevertheless, estradiol treatment following ovariectomy was still able to reduce LH levels (82). Estradiol injection failed to induce the LH surge in KERKO animals indicating a loss of estradiol positive feedback (82). In addition, KERKO mice lost the LH response to kisspeptin and showed a decreased LH response to GnRH injections (85). Along with these findings, electrophysiological studies demonstrated that RP3V kisspeptin cell activity was increased during estradiol positive feedback in control animals, but to a lesser extent in KERKO females (85). In contrast, ARC kisspeptin neurons activity was reduced during estradiol positive feedback in controls, but instead increased in KERKO females. These results were further confirmed using calcium imaging with protein-based indicators (GCaMP) fiber photometry approach, where KERKO mice showed an increased ARC *Kiss1*-expressing cell activity compared with intact controls, that was similar to ovariectomized controls (84). Thus, in females, ERα is required in kisspeptin neurons for complete maturation of the HPG axis and control of estradiol negative and positive feedback on GnRH/LH secretion. Although not studied in detail, the authors mentioned that KERKO males did not show modification in testicular weight or alterations in the LH response to castration (83), suggesting that ERα in kisspeptin neurons is not indispensable for estradiol regulation of male HPG axis.

KERKO animals have *Esr1* deleted in both RP3V and ARC kisspeptin neuronal populations. The role of ERα in KNDy neurons of the ARC was studied using a mouse model deleted for *Esr1* in *Tac2*-expressing cells, by crossing *Tac2* ^Cre^ mice with *Esr1* ^flox^ animals (ERα^Tac2^KO (81)). Nearly all *Tac2*-expressing cells co-express ERα. ERα^Tac2^KO females presented morphological abnormalities in ovaries and uteri, precocious puberty and impaired cyclicity. Basal LH levels were elevated in mutant animals compared to control showing an impairment of estradiol negative feedback (81). The role of this cell population in the estradiol positive feedback needs to be further detailed. It is important to note that *Kiss1* has been detected in ovaries and *Tac3* in both pituitary and ovaries (86–88). Thus, it is likely that in these KERKO and ERα^Tac2^KO models, *Esr1* was deleted in some cells outside the brain.

*Esr1* is also expressed in GABAergic and glutamatergic neurons in key hypothalamic regions including the RP3V and the ARC. These neurons are known to project directly to GnRH neurons. In females, mouse models deleted for *Esr1* in GABAergic neurons (*Vgat*-ires-Cre;*Esr1* ^lox/lox^) showed no modification of the age of puberty onset or estradiol regulation of the negative feedback. Indeed, basal LH levels were unchanged and mutant mice responded similarly to control to ovariectomy and estradiol replacement. However, *Vgat*-ires-Cre; *Esr1* ^lox/lox^ females presented abnormal cyclicity, infertility and did not show the normal estradiol positive feedback rise in LH (89). In males, deletion of *Esr1* in GABAergic neurons did not modify testicular weight, serum testosterone levels or fertility compared to controls, suggesting that this population is not necessary for proper functioning of the male HPG axis (90).

In parallel, the deletion of *Esr1* in glutamatergic neurons in females (*Vglut2*-ires-Cre;*Esr1* ^lox/lox^) induced advanced puberty onset, disturbed cyclicity and infertility. Mutant females showed an impairment of estradiol negative feedback with elevated basal LH levels compared to control and abnormal response to ovariectomy and estradiol replacement. In addition, *Vglut2*-ires-Cre;*Esr1* ^lox/lox^ females were unable to present an LH surge from estradiol injection, illustrating a lack of estradiol positive feedback in these animals (89). Although studied with less detail, *Vglut2*-ires-Cre;*Esr1* ^lox/lox^ males were fertile, showed normal testicular weight but with a significant increase in serum testosterone levels compared with control animals, suggesting a role for this neuronal population in the control of negative feedback in males (90). These results highlight the existence of strong sex differences in the role of *Esr1* in GABAergic and glutamatergic neuron for the control of the HPG axis.

#### 3.1.3 Models of region-specific and time-dependent *Esr1* deletion

The genetic models discussed above all have the caveat that ERα was deleted early during development, and thus did not allow for a distinction between developmental (organizational) and adult (activational) roles of this receptor. In addition, these models did not target a specific region, resulting in a lack of precision in understanding the neural network. Targeted viral vector injection in adulthood has permitted the reduction of *Esr1* expression in a hypothalamic nucleus-specific and time-dependent manner.

Ablation of *Esr1* specifically in the ARC has been achieved by stereotactic injection of an adeno-associated virus containing Cre recombinase (AAV-Cre) specifically into ERα^flox/flox^ mice (91). The deletion of 60–90% of ERα-expressing cells in the ARC induced a disturbance in ovarian cyclicity. Basal LH level in mutant mice was similar to those in control females, but the increase in LH after ovariectomy was reduced, suggesting an activational role of ERα-expressing cells in the ARC for estradiol negative feedback (91).

More recently, the combination of CRISPR-Cas9 technique with targeted injection of viral vectors has enabled the deletion of *Esr1* in RP3V or ARC kisspeptin-expressing cells in adult females (AAV-*Esr1*) (84, 92). *Esr1* deletion in RP3V kisspeptin neurons in adulthood did not modify estrus cyclicity but blunted the estradiol-induced LH surge confirming the important role of this neural population in the control of estradiol positive feedback. This effect was linked to a decrease in electrophysiological excitability of RP3V kisspeptin neurons in RP3V-AAV-*Esr1* females compared to controls (92). Using the same technique, *Esr1* was deleted, in adulthood, in ARC kisspeptin neurons (ARC-AAV-*Esr1*). Depending on the study, these females showed either disrupted (92) or normal estrous cyclicity (84). A reduced LH response to kisspeptin or GnRH treatment compared to control was observed in Wang et al. (2019). Variability of effects was observed with regards to negative feedback regulation. No modification of LH pulse frequency or basal LH levels was observed between ARC-AAV-*Esr1* and control females (92). In this study, CRISPR-mediated *Esr1* knockdown was achieved in 60% of cell population. Mc Quillan et al. (2022) observed that the phenotype of the animals depended on the efficacy of the knockdown generated by CRISPR technology. Indeed, an *Esr1* knockdown greater than 70–80% in ARC kisspeptin neurons was necessary to generate an increased pattern of synchronization similar to that observed in control animals after ovariectomy (84). Thus, the high percentage of *Esr1* deletion required to alter LH secretion could explain the difference between the effects observed in these two studies. These results show that ERα in RP3V and ARC kisspeptin cells has activational roles in regulating estradiol positive and negative feedback, respectively.

### 3.2 Role of ERβ in the regulation of the HPG axis

ERβ, unlike ERα, is expressed in GnRH neurons. In addition to the indirect regulation of the HPG axis via ERα-expressing neurons, in particular kisspeptin neurons, estradiol could act directly on GnRH neurons via ERβ.

#### 3.2.1 Models of ubiquitous *Esr2* deletion

The two first models of ubiquitous deletion of *Esr2* were obtained by homologous recombination by targeting exon 3 of *Esr2* (66, 93). ERβKO males had normal urogenital tract, testicular function and spermatogenesis and were fertile (66, 93). Nevertheless, castrated ERβKO males showed higher LH levels than wildtypes indicating a role for ERβ in estradiol negative feedback (94). In females, ERβKO induced variable reproductive phenotypes ranging from mild hypofertility to complete infertility (66, 93). Genital tracts of ERβKO females were similar to control animals (66). Adult ERβKO ovaries were macroscopically normal and had normal antral follicles, but with fewer corpora lutea than controls, suggesting less efficient ovulation due to impaired ovarian function (66, 93). Ubiquitous deletion of *Esr2* had no major impact on female positive and negative feedbacks exerted by estradiol. Basal LH levels of ERβKO females were either normal (73) or slightly increased (74). Treatment of ovariectomized females with estradiol resulted in a similar induction of the LH surge in both control and mutant mice (69). Furthermore, the distribution and number of GnRH neurons were unchanged and the opposite estradiol regulation of kisspeptin expression in the RP3V and ARC was maintained in ERβKO mice (38, 69). ERβ appeared, however, to be involved in the rapid action of estradiol on GnRH neurons. Rapid modulation of the phosphorylation state of the cAMP response element-binding protein (CREB) in GnRH neurons was abolished in mutant females (95). Importantly, these models were later shown to still express transcripts from alternative splicing of *Esr2* (53), which could be the cause of these variable fertility phenotypes. Therefore, a line devoid of any *Esr2* transcripts was generated using the Cre/loxP technique (named ERβ_ST_^L−/L−^ (96)). Mutant males and females from this model showed complete infertility. In females, it was due to drastic impairment of cyclicity and ovarian function. In males, the reason was unclear, as testis and epididymis histology and apparent motility of spermatozoa appeared normal (96).

#### 3.2.2 Models of brain specific *Esr2* deletion

ERβ is highly expressed in both female and male urogenital tracts, including the ovary, prostate and epididymis (97, 98). Thus, similarly to ERα, the infertility induced by the complete and ubiquitous deletion of *Esr2* did not allow the study of its neural effects. Although fewer in number than for ERα, more restrictive approaches have been used.

A mouse model selectively deleted for *Esr2* in the forebrain and pituitary has been generated by crossing *Esr2^flox^* mice (96) with CamKIIα-Cre mice (79). Mutant females showed normal cyclicity, basal LH levels and a normal increase in LH level after ovariectomy. However, their inability to reduce LH secretion after acute estradiol injection suggested a contribution of forebrain/pituitary ERβ in acute negative feedback of estradiol (79).

The role of neural ERβ was studied using an ERβ^NesCre^ mouse line (99). Mutant ERβ^NesCre^ females presented delayed puberty initiation with delayed vaginal opening and first estrus and delay of uterine growth (99). This pubertal delay was linked to a delay in postnatal expression of kisspeptin neurons in the RP3V. In adulthood, the number of GnRH neurons in the POA was unchanged, as was kisspeptin-immunoreactivity in the RP3V and ARC. Adult ERβ^NesCre^ females showed no change in estradiol levels, nor in LH response to ovariectomy, and had regular estrous cyclicity and fertility (99). The initiation of male puberty has not yet been evaluated in this model. In adulthood, ERβ^NesCre^ males were fertile, with seminal vesicles weights and testosterone levels unchanged from control littermates (100).

Selective *Esr2* deletion in GnRH neurons (GnRH-Cre;ERβ^loxP/loxP^) did not disrupt cyclicity, basal LH level, or LH response to estradiol. However, there was a reduced increase in LH secretion after ovariectomy in mutant mice compared to controls, suggesting a minor role for ERβ-expressing GnRH neurons in controlling estradiol negative feedback (79). Another study using a different mouse model observed a more drastic phenotype in GnRH-ERβ-KO (GERβKO) females, which exhibited ovarian morphology abnormalities, delayed puberty initiation, impaired fertility and reduced basal and surge LH levels without altering estradiol-negative feedback (101). While studied in less details, GERβKO males showed normal basal LH levels and no change in the timing of preputial separation (101). Although not totally clear, the use of different genetic background strains and knockout approaches could explain some of these discrepancies. Lastly, another study demonstrated that the rapid action of estradiol to phosphorylate CREB in GnRH neurons involved ERβ expressed by GnRH neurons themselves (102).

Overall, these genetic studies showed that neural ERα is essential for fertility and both positive and negative feedback controls exerted by estradiol on the HPG axis in females. These studies also suggest that neural ERα participates in the prepubertal regulation of the female HPG axis, necessary for its activation at puberty. The lack of studies in males makes it difficult to draw definite conclusions, although neural ERα does not appear to be essential for maintaining fertility in males. Its role in estradiol negative feedback remains to be clarified. Regarding neural ERβ, further work will be needed to understand the discrepancies observed in the different mouse models generated. Nevertheless, neural ERβ appears to be less critical for fertility than ERα, since it seems to play a more subtle role in regulating estradiol positive and negative feedback through both classical and non-classical signaling pathways. Interestingly, ERβ appears to play an activating role in puberty initiation, and additional studies will be crucial to understanding the underlying mechanisms of action.

## 4 ERα versus ERβ in estradiol-induced regulation of sexual behavior

Alongside their role in regulating the HPG axis, ERα and ERβ receptors are present throughout the neural circuits involved in the expression of sexual behavior (**Figure 2**). The specific involvement of either receptor in the regulation of sexual behavior by estradiol has been addressed in genetic studies presented below.

### 4.1 Role of ERα in the regulation of sexual behavior

The predominant expression of ERα in brain regions involved in the expression of sexual behavior, including the MeA, BNST, VMH, MPOA and ARC, has made it the primary target of research on ERs.

#### 4.1.1 Models of ubiquitous *Esr1* deletion

All studies carried out on ubiquitous ERαKO mouse lines showed a very strong disturbance of sexual behavior in both intact and hormonal-replaced males and females. ERαKO males show altered sexual behavior manifested by a sharp decrease in frequency of intromissions and an increase in latencies (103–106). In addition, no (106, 107) or little ejaculation (70, 103) was observed in ERαKO males. Different results have been reported regarding the number and latency times of mounts, which were found to be equivalent (103) or reduced (106) compared to wildtype mice. Various observations were also made regarding the role of ERα in regulating olfactory preference in males. ERαKO male mice were first shown to have similar interest in the odors of receptive female mice as controls (108). Other studies have observed altered olfactory preference for receptive females in ERαKO males compared to controls (71, 109, 110).

In ERαKO females, lordosis behavior was severely impaired compared to wildtype mice (63, 111–114). This effect was linked to a marked reduction in induction of progesterone receptor (PR) expression by estradiol in the VMH of mutant females (113). In addition, ERαKO females exhibited a strong deficit in proceptive behavioral interactions. While the number of mount attempts by males was similar between mutant and wildtype females, ERαKO mice vigorously rejected mounts from males that were unable to intromit (112, 114). However, stud males were equally attracted to ERαKO as to wildtype females during preference tests, suggesting that ERα was not critical to female attractivity (114). It is important to note that ubiquitous *Esr1* deletion induced elevated levels of estrogen and testosterone in mutant animals compared to wildtypes, which may affected brain functions through developmental processes (70, 71, 115).

The role of ERα in ERE-independent mechanisms of estradiol was investigated using the ERα^-/AA^ mice model. In this model, ERα signaling through ERE binding mechanisms was abolished (76, 77). ERα^-/AA^ males showed a strong deficit in sexual behaviors suggesting that ERE-independent ERα signaling was not sufficient to maintain sexual behavior in males and that genomic action of estradiol is critical (77). Another model harboring a mutation of the ERα palmitoylation site, which prevents membrane ERα signaling, showed no change in female sexual behavior but a strong reduction in male sexual behavior, suggesting an involvement of ERα membrane-initiated estrogen signaling in the organization of male sexual behavior (116).

#### 4.1.2 Models of brain specific *Esr1* deletion

To understand the specific role of neural ERα in the regulation of sexual behavior, several studies have used more restricted deletion models.

Sexual behavior was assessed in both ERα^NesCre^ males and females (80). Female sexual behavior, under normalized hormonal levels, was completely abolished with mutant females never exhibiting lordosis posture in response to male mounts. This impairment was linked with a drastic decrease in the number of progesterone receptor (PR)-expressing cells in the VMH of mutant animals compared to control littermates. In males, neural *Esr1* deletion induced a less severe behavioral phenotype (80). Mutant males were able to emit courtship vocalizations although with reduced number and duration of syllables. They also initiated mounts, intromissions and reached ejaculation, but with disturbance in the numbers and latencies of the events, provoking a less effective sexual behavior compared to control littermates and to mutants lacking both neural *Esr1* and *Ar* (80). Olfactory preference tests showed that both ERα^NesCre^ females and males presented similar preference toward males or receptive females as control littermates, suggesting an effect of *Esr1* deletion downstream the olfactory system (80). In particular, immunohistochemical analyses showed feminized calbindin and tyrosine hydroxylase neuronal populations of the mPOA, suggesting an implication of ERα in the perinatal organization of the neural circuitry underlying male sexual behavior (80).

To determine the role of ERα in either excitatory (glutamatergic) versus inhibitory (GABAergic) neurons, *Vglut2*-Cre;*Esr1* ^lox/lox^ and *Vgat*-Cre;*Esr1* ^lox/lox^ males were subjected to sexual behavior phenotyping (90). To date, female sexual behavior has not been tested in these models. In adult males, ERα-expressing neurons are mostly GABAergic in the mPOA, BNST and posterior dorsal MeA while they are predominantly glutamatergic in the VMHvl and posterior ventral MeA. Analysis demonstrated that the percentage of animals displaying mounting, intromission and ejaculation behaviors during the 30 min test was unchanged between *Vglut2*-Cre;*Esr1* ^lox/lox^ and wildtype males. In contrast, *Vgat*-Cre;*Esr1* ^lox/lox^ males showed similar mounting and intromission behaviors, but with a deficit in the ability to ejaculate, with only 25% of mutant males reaching it compared to controls, suggesting a role for GABAergic signaling in controlling male sexual behavior (90).

#### 4.1.3 Models of region-specific and time dependent *Esr1* deletion

Further studies addressed the developmental versus activational roles of ERα and the hypothalamic nuclei involved.

In males, using viral mediated RNA interference technique, deletion of *Esr1* specifically in adulthood or during prepuberty, in the mPOA or VMH, altered components of male sexual behavior illustrated by a reduced number of mounts and intromissions during the 30-minute test (29, 117). In contrast, adult deletion in the MeA did not affect these behaviors, while prepubertal deletion resulted in a reduced number of mounts and intromissions in adulthood. Ejaculation was not analyzed in this paradigm (29, 117). These results suggested that ERα within the mPOA and VMH is essential for the activational role of estradiol on male sexual behavior, whereas ERα in the MeA may not be crucial in adulthood but is necessary for the organization of the neural circuits underlying sexual behavior during prepuberty. Going further, calcium recordings of *Esr1*+ neurons activity in the mPOA, using fiber photometry, confirmed that mPOA-*Esr1+* neurons were activated during male mounts. Optogenetic stimulation of these neurons elicited mounting behavior, while the inhibition reduced it (118, 119). mPOA-*Esr1*+ neurons are mostly GABAergic (80%), and specific optogenetic activation of mPOA-*Esr1*+-*Vgat*+ neurons increased the mounting response compared to activation of all mPOA-*Esr1*+ neurons (119). Importantly, in both studies, activation of mPOA-*Esr1*+ or mPOA-*Esr1*+-*Vgat*+ neurons promoted male-typical mounting behavior also in females suggesting that female adult mPOA maintains the functional neural circuits to execute this behavior (118, 119). Thus, it is suggested that sexually dimorphic activation of mPOA-*Esr1*+ cells would underlie sex differences in the expression of reproductive behaviors. Indeed, as detailed in section 5, the activation of mPOA-*Esr1*+ cells also regulate female-typical pup retrieval behavior.

Regarding the role of the VMH in regulating male sexual behavior, additional studies showed that unlike the mPOA, adult inhibition of the VMHvl or VMHvl-*Esr1*+ neurons did not disrupt mounting behavior (120). This suggests that while the mPOA is critical for the expression of male-typical mounting behavior, the VMH may contribute but is not necessary. Although defensive behaviors are not the focus of this review, in males the VMH and especially ERα expressing neurons in this region have been shown to control aggressive behavior (119–121).

Another region known to control sexual behavior is the BNST, a major upstream region of the mPOA and the VMH. *Esr1*+ neurons of the principal subdivision of the BNST (BNSTpr) were chemogenetically silenced during ongoing social behaviors. The results showed reduced male mounting behavior towards a female when silencing took place during the approach phase, but this reduction was not observed during female mounting (122). However, silencing during the attack interrupted this behavior. Going further with optogenetic silencing of BNST projections, the authors showed that the appetitive phase of male sexual behavior (sniffing to mount) depended primarily on the activity of BNSTpr-mPOA projections, while aggression was triggered by BNST-VMHvl projections. BNST is also a node for olfactory sex recognition, with optogenetic inhibition of BNST-*Esr1*+ neurons reducing olfactory preference for females over males (122, 123). Interestingly, single-cell calcium measurements showed that different BNST-*Esr1*+ cell populations responded to male versus female stimuli. The same was observed in the mPOA and VMH-*Esr1*+ populations. BNSTpr-*Esr1*+ neurons were therefore found involved in controlling the transition between the appetitive and consummatory phases of male sexual behavior (122).

In females, the ablation of *Esr1* specifically in the VMH, in adulthood, was performed by RNA interference, using AAV vectors encoding for small hairpin RNA (shRNA) targeting the *Esr1* gene. An 80% reduction in *Esr1* expression abolished proceptive as well as lordosis behaviors in female mice (124) and rats (125), in association with a reduced estradiol-induced PR expression in this region (124). In the VMH, the neurons expressing *Pgr* (coding for PR) co-express *Esr1*. This neuronal population has been shown to project to the RP3V, the POA and the PAG (126). Interestingly, projections to RP3V showed strong structural plasticity, with more projections in females in estrus stage than in diestrus, suggesting a role for this neuronal population in linking sexual behavior and ovulation. This hormone-driven change was not observed for projections to the POA or PAG. Going further, using electrophysiological and optogenetics studies, the authors showed that these VMH-RP3V projections were mostly glutamatergic, and that ovarian sex hormones enhanced these excitatory projections by increasing the number of glutamatergic synapses formed into RP3V neurons (126).

Regarding the role of MeA or POA in female sexual behavior, the suppression of ERα in the MeA of female rats did not show any effects (125), but the reduction of *Esr1* expression specifically in the mPOA increased lordosis behavior in mutant females compared to controls, without modification of proceptive behaviors (127). These results suggested that ERα is involved in the inhibitory action of the mPOA on lordosis behavior. Surprisingly, a study in female mice showed that reduction of *Esr1* expression in the mPOA induced a small decrease in receptive behaviors compared to control (128). However, these experiments were performed on gonadally intact female mice that expressed low levels of lordosis behaviors, making it difficult to compare the estradiol-induced effects between control and mutants.

### 4.2 Role of ERβ in the regulation of sexual behavior

Although ERβ is less expressed than ERα, it is found in most regions involved in the expression of sexual behavior. Fewer studies have investigated its function in the regulation of sexual behavior.

#### 4.2.1 Models of ubiquitous *Esr2* deletion

Behavioral characterization of the first lines of ERβKO mice (93) showed no disturbance in any aspects of male (latency and number of mounts and intromissions) or female sexual behaviors (lordosis quotient) evaluated at adulthood (129, 130). Olfactory preference was also unaffected in ERβKO males (130). Nevertheless, a role for ERβ in the defeminization of the brain has been suggested by the increase in female-like lordosis behavior displayed by adult ERβKO males when primed with estradiol and progesterone (130). In addition, a delay in behavioral pubertal maturation was observed, with ERβKO males showing a delay in the age of first ejaculatory behavior (94). Interestingly, ERβKO displayed elevated testosterone levels in pubertal and young adults (5–12 weeks), not found in older mice, supporting a potential role for ERβ during pubertal development (131). It is important to note that in this ERβKO mouse line, some *Esr2* transcripts were still present. Different results were observed using the genetic model devoid of all *Esr2* transcripts (ERβ_ST_^L−/L−^ (96);). Indeed, in this model, mutant males exhibited mild alteration of sexual behavior, with an increase in the number of mounts and intromissions and in the latency to ejaculate compared to controls. Differences observed between controls and mutants declined with sexual experience (132). In females, ERβ_ST_^L−/L−^ mice showed reduced attractiveness and lordosis behavior (132).

#### 4.2.2 Models of brain specific *Esr2* deletion

The first studies were carried out in female rats, in which adult intracerebroventricular administration of an *Esr2* antisense oligonucleotide showed no effect on lordosis behavior (133). Then, the role of neural ERβ in regulating sexual behavior was evaluated using ERβ^NesCre^ males and females (99, 100). In males, neural *Esr2* deletion had no impact on the expression of sexual behavior, with mutant animals displaying the full range of sexual behaviors and achieving ejaculation similarly to wildtypes (100). No change in olfactory preference was observed either. In contrast to what was observed in ubiquitous ERβKO males, the analysis of female-like lordosis behavior in castrated ERβ^NesCre^ males primed with estradiol and progesterone did not reveal any significant change between mutants and controls (100). Regarding the females, ERβ^NesCre^ animals displayed unchanged olfactory preference and lordosis behavior compared with controls (99). These results showed that neural ERβ is not necessary for the proper expression of male and female sexual behaviors at adulthood.

#### 4.2.3 Model of region-specific and time dependent *Esr2* deletion

Few studies have gone further in discriminating between potential developmental versus activational roles of ERβ, and hypothalamic neural pathways involved.

In males, site-specific knockdown of ERβ in MeA and mPOA was obtained by injecting small-hairpin RNA (shRNA)-associated adenoviruses (AAV) into these regions during prepuberty (PND21) or at adulthood (134). Prepubertal or adult silencing of *Esr2* in mPOA or MeA did not alter the latencies and numbers of mounts and intromissions in adult males. An alteration in olfactory preference towards receptive versus non-receptive females was observed with adult *Esr2* silencing. No alteration was observed when tested for preference between a receptive female and a male (134). Going further, fiber photometry recording did show higher activity of MeA-ERβ+ cells when sniffing a receptive female than when sniffing a non-receptive one or a gonadally intact male. Interestingly, chemogenetic inhibition of these neurons abolished the preference for a receptive female over a non-receptive female, but did not change the preference for a receptive female over a male. The authors also identified that MeA-ERβ+ neuronal projections to the BNST participate in this olfactory preference based on female receptivity but not on sex (135). In the MeA, ERβ appeared to be involved in distinguishing receptive states of female mice.

Very few studies have been done in females. In rats, the silencing of ERβ by injection of an shRNA-associated AAV in the VMH, did not modify the lordosis response (136). In addition, in female mice, injection of shRNA-associated AAV into the dorsal raphe nucleus to specifically knockdown ERβ in adulthood, did not modify the lordosis response on the day of estrus, but led to a sustained lordosis response on the day following behavioral estrus (137). The same observations have been made previously in ubiquitous ERβKO females, suggesting a role for ERβ in the inhibitory regulation of female sexual behavior outside the estrus phase (129).

Collectively, these genetic studies have demonstrated the crucial role of neural ERα in regulating both male and female sexual behavior. Its roles and mechanisms of action are extremely complex and depend on sex, age and brain region. Recent elegant studies, using a combination of innovative techniques, have paved the way for establishing the precise neural pathways at play in the control of these behaviors. Fewer studies have focused on the role of ERβ, as the data indicate a less important role for this receptor in the control of sexual behavior. Indeed, ERβ appears to be more involved in the control of other social and mood related behaviors (99, 138). Nevertheless, studies suggest that ERβ participates in the pubertal organization of neural circuits involved in the control of sexual behavior, at least in males (94, 131). Further studies will be instrumental to go deeper into the mechanism of action and establish whether the same is true in females.

## 5 Estradiol regulation of parental behavior

In addition to their role in regulating sexual behaviors, several neural regions that express ERα and ERβ are also known to control parental behavior, especially the POA, VMH, BNST, and MeA. Parental behavior is crucial for species survival and offspring development (**Figure 3**). These brain regions exhibited an increase in *Fos* expression, a marker of neural activation, in response to the display of maternal behavior. Between 25% and 45% of these *Fos+* immunoreactive cells co-expressed ERα, suggesting that maternal behavior possibly involved neural ERα activity (139). Furthermore, natural variations in the level of maternal care were associated with changes in *Esr1* expression, but not that of *Esr2* (140). This suggests a potential role of ERα in regulating maternal behavior, which was further investigated in the genetic studies described below. To date, no genetic studies have addressed the role of ERβ in the expression of parental behavior.

### 5.1 Role of ERα in the regulation of parental behavior

#### 5.1.1 Models of ubiquitous *Esr1* deletion

Studies using ubiquitous ERαKO animals were performed in mice (104, 111, 112) and more recently in rats (141, 142). Due to the infertility of ERαKO animals, analysis of parental behavior was carried out in nulliparous animals following a pup sensitization process, which involves repeated exposure of the animals to pups produced by donor lactating mothers. Maternal behavior of ERαKO mice was strongly disturbed in both intact and gonadectomized females. Mutant females displayed impaired pup-retrieving behavior and a high level of infanticide (111, 112). In male mice, ubiquitous *Esr1* deletion had no effects on pup retrieval, but ERαKO males showed a high percentage of infanticide, that was abolished after gonadectomy (104). The authors suggested that the increase in testosterone levels observed in ERαKO males could be sufficient to promote infanticide, by acting on AR. Surprisingly, in rats, juvenile and adult ERαKO females showed no modification in maternal behavior. The latency score for retrieving and regrouping the pups and adopting a crouched position over them was identical in mutant and control animals (141, 142). Only a slight impairment was observed when the experimental tests were performed in a novel cage (142). The discrepancy with the data obtained in mice is not yet understood, but could result from different underlying mechanisms controlling maternal behavior in the two species. Moreover, deletion strategies diverged between the two ERαKO animal lines, with homologous recombination targeting exon 2 in mice (63, 143), and zinc finger nucleases (ZFNs)-mediated genome editing targeting exon 3 in rats (144). In juvenile male rats, ubiquitous *Esr1* deletion did not alter the pup retrieval latency score compared with controls. The level of infanticide was not analyzed in these animals (141).

The discrepancies observed in these models underline the need to conduct genetic research under physiological conditions and to use more targeted approaches of *Esr1* deletion in the neural regions of interest. Maternal behavior was only studied in nulliparous females, which did not experience the hormonal changes that occur during gestation and parturition in preparation for behavioral processing. Moreover, males and females lacked stimulation of the ERα signaling pathway during embryonic, postnatal and pubertal development periods, which could interfere with the organization of the neural pathways underlying parental behavior.

#### 5.1.2 Models of region-specific and time dependent *Esr1* deletion

Over the last decades, a growing number of studies have focused on region-specific deletions of *Esr1*, with particular emphasis on the mPOA, the main region controlling parental behavior.

In female mice, reduction of *Esr1* expression in the mPOA, via targeted viral-vector mediated siRNA silencing, significantly diminished pup retrieving behavior in sexually naive females (128). A similar behavioral impairment was observed when mPOA-*Esr1*+ cells were ablated (118) or chemogenetically inhibited (145) in naive and lactating females. In addition, experiments performed optogenetic inhibition of mPOA-*Esr1*+ neurons at specific time points in the behavioral sequence. This study demonstrated the critical role of these neurons in promoting pup contacts once females have initiated pup approach, and in facilitating the completion of pup retrieval once females have initiated this behavior (118). However, inhibition of these mPOA-*Esr1*+ neurons after the females had initiated crouching over the pups had no effect on this behavior. Conversely, optogenetic activation of mPOA-*Esr1+* cells increased pup retrieval in both naive and lactating females (118, 145). With regards to the expression of other maternal behaviors, the reduction in *Esr1*+ expression in the mPOA decreased the time the female spent licking and nursing the pups without affecting maternal aggression (128). Fang et al., 2018 also reported that chemogenetic inhibition of mPOA-*Esr1*+ cells had no effect on sniffing, grooming, and crouching over the pups (145).

In the mPOA, galanin-expressing neurons (mPOA-*gal*) are crucial for the expression of parental behavior in mice (146, 147). They represent about 20% of mPOA neurons, and the majority of them express ERα. A recent study showed that AAV-mediated ablation of *Esr1* in mPOA-*gal* neurons drastically reduced the expression of pregnancy-induced maternal behaviors, including intensive nest building and pup retrieval behaviors, that occur from the first days of gestation. The parental behavior of these animals remained impaired after birth, indicating that these effects could not be compensated for by the effect of the hormonal milieu during parturition (148). Interestingly, the authors also demonstrated that estradiol play a role in the neural modeling of the mPOA-gal neurons during pregnancy, by transiently silencing mPOA-gal neurons and contradictively increasing their excitability. The deletion of *Esr1* in mPOA-*gal* neurons prevented this remodeling (148). These results indicated that within the mPOA, ERα signaling appears to facilitate pup care behaviors.

Other studies have focused on other parameters of parental behavior, such as increased interest in the pups leading to a reduction in infanticide, while there is an increased aggressivity towards intruders. In this context, Mei et al. (2023) uncovered a neural circuit between the BNSTp and the mPOA that modulates the display of female infanticide towards pups (149). Indeed, although the mechanism underlying this state-dependent switch remains unclear, sexually naive females often kill their pups, while lactating females show maternal care. Interestingly, chemogenetic inhibition of mPOA-*Esr1+* cells projecting to the BNSTp increased pup attacks by females, while chemogenetic activation of these cells reduced infanticide. On the contrary, blocking the input from BNSTp*-Esr1+* to the mPOA suppressed infanticide, while activating this projection suppressed maternal behavior. Furthermore, *in vitro* current-clamp recording showed that BNSTp-*Esr1*+ neuronal population switched from excitable in naive females to less excitable in lactating dams, in contrast to mPOA-*Esr1*+ cells which became more excitable in lactating dams than in naive females (149). These results support the existence of a neural circuitry remodeling between mPOA and BNSTp during motherhood that is dependent on estrogen signaling. In particular, there appears to be reciprocal inhibition between these two regions to control the levels of infanticide versus maternal behavior.

On the other hand, females typically display low levels of aggression, except during gestation and lactation, when dams show high levels of maternal aggression towards perceived threats to their offspring (150). This behavior is displayed from the first days of gestation, in response to the hormonal changes occurring with pregnancy. The ventrolateral part of the VMH (VMHvl) has been identified as a key hypothalamic region controlling maternal aggression in females. The same region has been extensively studied for its role in territorial aggression in males (151). Importantly, the role of VMHvl neural population in controlling female aggression is dependent on the genetic background and the reproductive state of the animals. Focusing on experiments using lactating females only, it has been showed that VMHvl-*Esr1+* cells are critical for maternal aggression. Indeed, using the GCaMP fiber photometry approach, it was shown that VMHvl-*Esr1*+ cells were activated during attacks by a lactating female towards a juvenile or adult male intruder, but not when the lactating female was investigating or retrieving pups. Moreover, inhibition of VMHvl-*Esr1*+ cells in these mice drastically decreased aggression against intruders (152). Further studies, described below in 6., have used omics analyses to identify specific neural populations located in the POA and VMH which mediate maternal behaviors, and have differentiated them from cell clusters specifically activated during sexual behavior.

Collectively, these studies provide evidence that ERα regulates maternal behavior in mice. Within the mPOA, ERα appears to facilitate pup care behaviors, while it seems implicated in regulating infanticide in the BNSTp, and maternal aggression in the VMHvl. Because mouse strains used in laboratory experiments are a monoparental species, the investigation of paternal behavior remains very limited. One study in male mice showed that ablation of mPOA-*Esr1+* neurons had no effect on pup retrieval behavior, whereas optogenetic activation of these cells induced this behavior (118). These results suggested that unlike in females, mPOA-*Esr1*+ neurons may not be necessary for the display of pup retrieval in males. Nevertheless, additional studies are needed to establish the role of estradiol in the regulation of paternal behavior, especially using biparental species like prairie voles or Californian mice.

### 5.2 Lack of models of *Esr2* deletions

No genetic study has addressed the role of ERβ in the regulation of parental behavior. Yet this receptor appears to be tightly associated with oxytocin, a key hormone that facilitates maternal behavior. It has been shown that estrogen treatment increased oxytocin gene expression in the rat paraventricular nucleus (153). Oxytocin neurons in this region were found essential for the regulation of emotional and social behaviors, including parental care (for reviews: Acevedo-Rodriguez, Mani & Handa, (154); Neumann, (155)). In the paraventricular nucleus, approximately 84% of oxytocin neurons co-express ERβ, a colocalization not observed in other oxytocinergic regions such as the supraoptic nucleus (156). Estradiol treatment also increased oxytocin expression in the paraventricular nucleus of both male and female mice. This effect was not observed in ERβKO animals (157, 158). These data highlight the need for genetic studies to unravel the implication of ERβ in the regulation of parental behavior, notably in relation to oxytocin.

## 6 Molecular profiling of brain circuits controlling sex- and reproductive state- dependent reproductive behaviors

In recent years, several innovative techniques have allowed the identification of specific neural populations that control especially male versus female behaviors in the main regions controlling reproductive behaviors (POA, VMH, BNST and MeA).

Combination of single-cell RNA-seq (scRNA-seq) and multiplexed error-robust fluorescence *in situ* hybridization (MERFISH) analysis of the POA allowed the identification of 70 transcriptomic cell types (T-types), including T-types preferentially activated in females or males during specific social behaviors, particularly parenting, aggression, and mating (159). In this study, by including fos probes in MERFISH measurements, it was shown that cell clusters activated by parenting and mating (i.e. enriched in fos-positive cells) appeared to belong to two transcriptionally distinct cell populations localized into distinct preoptic nuclei (159). Interestingly, *Esr1* expression has been found in nearly all behaviorally activated clusters. With regard to mating, it was shown that some clusters enriched in fos-positive cells were activated in both sexes after mating, while a few clusters exhibited sexually dimorphic activation (159).

Several clusters expressing *Esr1* in the VMH have also been found to be enriched in a sexually dimorphic manner. For example, *Esr1*+ cells located in the VMHvl have been shown to co-express PR and control especially female sexual behavior and male aggression (120, 160–162). In males, calcium imaging analysis of VMHvl-*Esr1*+ neurons showed distinct cell populations activated by male versus female stimuli during resident-intruder assays. Intriguingly, these differences appeared with social and sexual experience, since in naive animals, male and female intruders activated overlapping neuronal populations (163). Going further into synaptic connectivity, viral-genetic tracing in *Esr1*-Cre male and female mice revealed that most inputs and outputs of VMHvl-*Esr1*+ neurons were located in the hypothalamus and extended amygdala with a high degree of bidirectional connectivity (164). scRNA-seq analysis identified 17 different sub-types of cells in the VMHvl, including seven *Esr1*+ T-types (162). Among them, the authors identified two anatomically distinct subsets of VMHvl-*Esr1*+ neurons, which preferably project to dPAG or mPOA, and would therefore play a role in the control of behavior, or provide feedback to hypothalamic and amygdala circuits, respectively (162, 164). A female-specific cluster has also been identified and subsequently confirmed to be specifically activated during mating (162, 165). Indeed, in females, a reproductive state-dependent switch in female behavior has been revealed, with molecularly distinct subpopulations of VMHvl*-Esr1*+ neurons excited during maternal aggression or sexual behavior (152, 165). The use of activity-dependent single-cell RNA sequencing allowed the identification of VMHvl T-types specifically activated in either lactating females exhibiting attack or virgins exhibiting lordosis (165). Among these clusters, using several optogenetic manipulations, the authors identified two transcriptomically distinct VMHvl-*Esr1*+ subtypes and were able to assign causal roles in mating versus aggression in virgins and lactating mothers, respectively. Interestingly, aggression-specific cells displayed changes in bulk-calcium activity depending on the reproductive status, showing an increase in response to social cues (male or female intruders) across the transition from virginity to lactation (165).

A broader approach used translating ribosome affinity purification and sequencing (TRAPseq), and scRNA-seq to identify differentially expressed genes (DEGs) between males and females (comprising females at two estrous stages, estrus and diestrus), specifically in *Esr1*+ neuronal population. These analyses were carried out in the BNSTpr, MeA, POA and VMHvl. The authors identified 1,415 DEGs between sexes and estrous stages, divided in 137 *Esr1*+ cell types (166). Almost all *Esr1*+ cell types co-expressed PR and AR, showing a strong hormonal influence on these populations. This study also confirmed that BNSTpr and POA are mostly GABAergic, the MeA composed of both excitatory and inhibitory neurons, whereas the VMHvl is largely glutamatergic. Nevertheless, a small population of *Esr1*+ inhibitory neurons was identified in the VMHvl (166). The authors focused on the behavioral role of two specific cell types.

First, BNSTpr-*Esr1*+-*Tac1*+ GABAergic neurons were found to be the most enriched in males compared with females, independently of their estrous stage. Chemogenetic inhibition of this population in adult males reduced sex recognition, mounting and intromission during mating, but also attacks during aggression. Interestingly, these effects were not observed when inhibiting BNSTpr-*Esr1*+-*Tac1*- neurons, showing the specific involvement of cells expressing both *Esr1* and *Tac1* in BNSTpr for the regulation of male reproductive behaviors (166). Further studies showed that BNSTpr-*Esr1*+-*Tac1*+ neurons project in the POA to POA-*Tacr1*+ neurons (167) and that these projections regulated male mating but not aggression. POA-*Tacr1*+ neurons co-express *Esr1*, and fiber photometry imaging showed that these neurons were activated during mounting and intromission toward females, but not activated during male-male attacks. The neuropeptide substance P encoded by the tachykinin 1 gene (*Tac1*), is the ligand for the tachykinin receptor 1 (Tac1r). Its release by BNSTpr-*Tac1*+ cells was shown to potentiate the activation of POA-*Tacr1*+ neurons and initiate male mating. The authors also revealed that POA-*Tacr1*+ projections to VTA and PAG control male sexual behavior (167).

Secondly, in females, the authors identified the VMHvl-*Esr1*+-*Cckar*+ glutamatergic cell type enriched in females in estrus compared to diestrus. Chemogenetic inhibition of this population highly reduced lordosis behavior without altering maternal behaviors, including pup retrieval and maternal aggression. An additional viral strategy showed that these VMHvl-*Esr1*+-*Cckar*+ neurons project preferentially to the AVPV and that these projections peak at estrus. These effects were specific to *Esr1*+-*Cckar*+ cells and were not observed in VMHvl-*Esr1*+-*Cckar*- neurons. Interestingly, there was no change in the number of projections to the PAG or POA, indicating a specific projection pattern for this neuronal population. In addition, inhibiting VMHvl-*Esr1*+-*Cckar*- neurons did not affect lordosis behavior in female but inhibited male mounting behavior and abolished maternal aggression (166).

Altogether these innovative techniques open the way to deciphering the specific neural pathways that control sexually dimorphic reproductive behaviors. Indeed, discrete cell populations appear to be involved in the activation/repression of specific behaviors, according to sex and hormonal state.

## 7 Discussion and perspectives

Survival of the species relies on the proper regulation of the HPG axis and optimal expression of sexual and parental behavior. These reproductive function and behaviors are strongly influenced by hormonal regulation, especially by estradiol. Extensive research has been carried out since the 1950s, leading to a better comprehension of the specific role of ERα and ERβ and their underlying mechanisms of action. Importantly, all this research points to the existence of an extremely complex neural circuit, which is regulated in different ways according to sex, hormonal status, and age. Recent technological progress has rendered increasingly possible the differentiation of these different parameters in order to obtain a precise view of the neural populations and neural circuits involved in these neuroendocrine and behavioral functions.

The data presented here show that neural ERα is mandatory for the activation and functioning of the gonadotropic axis, expression of sexual and parental behavior in females. In males, it plays a critical role, but it is not indispensable. Indeed, testosterone and its neural metabolite estradiol act in a complementary manner via both AR and ERs in males, with a crucial role established for AR (34, 80). The role of neural ERβ is not entirely clear, but it appears that it is not required for fertility and instead plays a more subtle role in modulating the HPG axis in adults of both sexes. Nevertheless, it is important to bear in mind that the majority of studies analyzing the role of estradiol in regulating the HPG axis have been carried out in females, and that further studies in males are needed to achieve a more thorough understanding. Evidence reported so far has revealed the important role of ERα signaling in the control of maternal behaviors. Further studies, especially in males, will be instrumental in gaining a better understanding of the estradiol regulation of parental behavior.

The recent studies monitoring the activity of *Esr1*+ cell populations during very specific reproductive behaviors, in different brain regions, as a function of sex and/or hormonal state highlight the existence of a strong neuroplasticity of the brain circuits controlling distinct reproductive behaviors. Interestingly, within the same hypothalamic nucleus, sexually dimorphic activation of different cell clusters corresponds to sexually dimorphic expression of behaviors. Further studies are needed to understand the role of estradiol and other sex steroids in the organization of these neural circuits. Indeed, most of the region-specific deletions have been carried-out in adulthood, but as techniques improve, it is becoming possible to make these genetic modifications at younger ages. It would be particularly interesting with regard to ERβ, whose role in adulthood seems more subtle, but for which several indications point to an implication during pubertal development. It is important to note that, while outside the scope of this review, ERβ is known to play important roles in estradiol regulation of emotional state and social behaviors (99, 138).

Finally, a better understanding of ERs signaling pathways in the regulation of reproductive function and behaviors has an important translational perspective. Indeed, human fertility is under the control of estrogens and *ESR1* and/or *ESR2* gene mutations and polymorphisms have been associated with reproductive defects in both men and women, including abnormal timing of puberty and infertility (168–175). Loss-of-function mutations in *ESR1* induce estrogen resistance in both men and women. The first men case reported normal pubertal development with normal male genitalia and sperm density (168). In addition, the patient had tall stature and delayed skeletal maturation and osteoporosis, with at adulthood a bone age of 15 years old. Indeed, while not in the scope of this review, it is well known that estrogen plays critical role in bone development and mineralization during puberty in both sexes. Testosterone concentrations were normal while estrogen and FSH and LH serum levels were increased. He indicated having sexual interests and had normal functioning, including morning erections and nocturnal emissions. The treatment with estrogen had no detectable effect (168). A loss-of-function *ESR1* mutation was identified in a woman without breast development, primary amenorrhea, a small uterus and multicystic ovaries (170). She also had elevated estrogen serum levels, mildly elevated gonadotropins and delayed bone age. Estrogen treatment did not change breast development but diminished ovarian and cyst size (170). Subsequently, few other *ESR1* mutations (2 females and 1 male) were identified describing similar clinical phenotypes (174). All suggested estrogen resistance syndrome and were in accordance with the reproductive phenotype of *Esr1* ubiquitous knockout mouse models described in section 4.1.1. *ESR2* mutations are even rarer. Monoallelic and biallelic *ESR2* variants have been identified in one syndromic and two nonsyndromic 46, XY patients with differences of sex development, presenting absent gonadal development or partial and complete gonadal dysgenesis. This suggested a role for ERβ in early gonadal development (175). In addition, a point mutation in *ESR2* was identified in a young woman with absent puberty without breast development, a small infantile uterus, no detectable ovaries and severe osteoporosis (173). In contrast to *ESR1*-deficient patients, estrogen levels were low. Estrogen and progestin replacement therapy enabled breast development, menarche and uterine maturation (173). These results suggest that *ESR2* is also necessary for human ovarian development and that *ESR1* is not sufficient to support ovarian function in human. *ESR1* and *ESR2* are expressed in human gonads, making it difficult to distinguish peripheral from central effects in these patients. Some studies have also associated *ESR1* and *ESR2* gene polymorphisms with infertility and assisted reproduction outcomes (171, 172). Further studies will be needed to understand the neural role of ERα and ERβ in regulating reproductive function in humans. The acquisition of greater knowledge could lead to the development of sex- and age-specific therapeutic strategies for fertility disorders, which represent a major public health issue as their frequency are increasing due to medical, environmental and societal causes.

## Author contributions

TT: Writing – original draft, Writing – review & editing. NA: Writing – original draft, Writing – review & editing. SMK: Writing – review & editing. LN: Writing – original draft, Writing – review & editing.
